# Timing of malaria in pregnancy and impact on infant growth and morbidity: a cohort study in Uganda

**DOI:** 10.1186/s12936-016-1135-7

**Published:** 2016-02-16

**Authors:** Pierre De Beaudrap, Eleanor Turyakira, Carolyn Nabasumba, Benon Tumwebaze, Patrice Piola, Yap Boum II, Rose McGready

**Affiliations:** Epicentre, Paris, France; Ceped, Institut de Recherche pour le Développement, Paris, France; Epicentre, Mbarara, Uganda; Mbarara University of Science and Technology (MUST), Mbarara, Uganda; Institut Pasteur de Madagascar, Tananarive, Madagascar; Shoklo Malaria Research Unit, Mahidol-Oxford Tropical Medicine Research Unit, Faculty of Tropical Medicine, Mahidol University, Mae Sot, Thailand; Centre for Tropical Medicine, Nuffield Department of Medicine, University of Oxford, Oxford, UK

**Keywords:** Malaria in pregnancy, Infant growth, Sub-Saharan Africa, Cohort

## Abstract

**Background:**

Malaria in pregnancy (MiP) is a major cause of fetal growth restriction and low birth weight in endemic areas of sub-Saharan Africa. Understanding of the impact of MiP on infant growth and infant risk of malaria or morbidity is poorly characterized. The objective of this study was to describe the impact of MIP on subsequent infant growth, malaria and morbidity.

**Methods:**

Between 2006 and 2009, 82 % (832/1018) of pregnant women with live-born singletons and ultrasound determined gestational age were enrolled in a prospective cohort with active weekly screening and treatment for malaria. Infants were followed monthly for growth and morbidity and received active monthly screening and treatment for malaria during their first year of life. Multivariate analyses were performed to analyse the association between malaria exposure during pregnancy and infants’ growth, malaria infections, diarrhoea episodes and acute respiratory infections.

**Results:**

Median time of infant follow-up was 12 months and infants born to a mother who had MiP were at increased risk of impaired height and weight gain (−2.71 cm, 95 % CI −4.17 to −1.25 and −0.42 kg, 95 % CI −0.76 to −0.08 at 12 months for >1 MiP compared to no MiP) and of malaria infection (relative risk 10.42, 95 % CI 2.64–41.10 for infants born to mothers with placental malaria). The risks of infant growth restriction and infant malaria infection were maximal when maternal malaria occurred in the 12 weeks prior to delivery. Recurrent MiP was also associated with acute respiratory infection (RR 1.96, 95 % CI 1.25–3.06) and diarrhoea during infancy (RR 1.93, 95 % CI 1.02–3.66).

**Conclusion:**

This study shows that despite frequent active screening and prompt treatment of MiP, impaired growth and an increased risk of malaria and non-malaria infections can be observed in the infants. Effective preventive measures in pregnancy remain a research priority.

This study was registered with ClinicalTrials.gov, number NCT00495508.

## Background

Malaria in pregnancy (MiP) remains a leading cause of miscarriage, preterm delivery, intrauterine growth restriction (IUGR) and low birth weight (LBW) in malaria-endemic areas of sub-Saharan Africa, Asia and South America [1–5]. As a result, MiP increases the risk of perinatal mortality as well as reducing the capacity of the child to develop to his/her full potential in these settings. The impact of MiP on infant growth and infant risk of malaria or morbidity, in contrast to adverse birth outcomes, is infrequently reported. Only two studies have investigated the relation between MiP and infant growth and found that placental or peripheral malaria at delivery was independently associated with lower weight at 12 months [6, 7]. Other studies have investigated the effect of MiP on the risk of malaria in infants but mixed results have been reported [8–15]. Some studies observed an increased risk of malaria in infants born to multigravida women with placental infection [9, 11], but this was not confirmed by others [10, 12]. Apart from the risk of malaria, there is a paucity of data on other morbidities commonly found in infants such as diarrhoea and pneumonia and on their association with MiP.

MiP may increase infant morbidity through different mechanisms. First, MiP is responsible for IUGR and preterm birth that are, in turn, associated with infant mortality and morbidity [16–19]. Also, there is increasing evidence showing maternal immune response and fetal immune system are in a highly dynamic state [20]. It has been suggested that the inflammatory response following placental malaria infection may lead to some immunological tolerance in the offspring [21, 22]. Moreover, the consequences of in utero infection may vary with maternal gravidity [11] and with the gestational age of exposure [23]. The accurate determination of both gestational age (GA) and weight at birth is, therefore, an important factor that should be included in the analysis of the effect of MiP on the risk of malaria infection in infant [24].

The objective of this work was to analyse the effect of MiP on infant growth and morbidity, (including malaria) with respect to MiP timing using data from a prospectively and previously published cohort of pregnant women and their infants [24]. This work differs to most previously published work from sub-Saharan Africa because GA was documented by ultrasound and the detection of malaria during pregnancy was active and frequent with regular screening at each visit with prompt treatment for mothers and infants.

## Methods

### Population and setting

This mother–baby cohort study was conducted in Mbarara district, southwestern Uganda. This predominantly rural area lies at an altitude of about 1500 m above sea level and has a tropical climate with a bimodal rainfall pattern averaging 1200 mm per annum in September–January and March–May. Malaria transmission was considered as mesoendemic although significant heterogeneity has been observed recently [25, 26].

### Study design

The study design was a prospective cohort of 1218 pregnant women of estimated GA ≥13 weeks with a nested clinical trial conducted between October 2006 and May 2009, in which 304 pregnant women were enrolled [27]. All women with a positive blood smear were invited to participate in a study comparing the efficacy and tolerance of artemether–lumefantrine (AL) with oral quinine for the treatment of uncomplicated falciparum malaria of whom 304 met the criteria and consented [27]. Newborns of mothers enrolled in the cohort were included in a birth cohort and actively followed until 12 months. Only live-born singletons with accurate GA estimation were included in this analysis.

### Clinical and monitoring procedures

At enrolment, a comprehensive assessment of the mothers was performed that included the collection of information on their demographic, socioeconomic, medical and obstetric characteristics, a clinical and obstetric examination, an ultrasound evaluation, blood smear and haemoglobin measurements. An estimation of GA by ultrasound for foetal biometry using biparietal diameter and femur length was performed between 16 and 22 weeks of pregnancy [28]. After the initial assessment, mothers were followed every week. Malaria infection was systematically screened using a Paracheck Pf^®^ (Orchid, Goa, India) rapid diagnostic test (RDT) and confirmed with a blood smear test.

Women in the cohort received standard supervised IPT with two doses of sulfadoxine-pyrimethamine (SP) given at intervals of one month or more during the second and third trimesters as recommended by national guidelines. IPT was not given to the women who receive anti-malarial treatment (quinine or AL). All treatments were provided free-of-charge.

At delivery, blood smears were obtained from the mother, the placenta, cord and from the newborn to verify the presence of malaria infection. Newborns were weighed to the nearest 10 g using a SECA mechanical type scale and received an initial standardized physical examination by a medical officer. Length was measured using a Stadiometer. After the initial evaluation, infants were seen every month or more frequently if required until 12 months. At each visit, anthropometric characteristics were measured once, malaria infection was screened with a Paracheck^®^ RDT and treated with AL, and a medical examination by a paediatrician was performed. Morbidity definitions in infancy were based on national guidelines [29].

### Laboratory procedures

Thick and thin blood smears were prepared and stained with Giemsa. Parasitaemia was calculated by counting parasites against 200 white blood cells. Placenta smears were taken by incising a fresh placenta on the maternal surface halfway between the cord and the periphery.

HIV testing and treatment was proposed for all women and performed according to the national guidelines.

### Definition

Small-for-GA (SGA) was defined as a birth weight less than the 10th percentile of sex-specific birth weight-for-GA [30]. Peripheral malaria infection was defined as the occurrence of a positive peripheral blood smear or rapid diagnostic test. Placental malaria was defined as the detection of any parasite in a placental blood smear by microscopy.

### Statistical analysis

#### Infant outcomes

The change in weight and height between birth and 12 months (weight and height gain) were analysed using a linear model. Missing data at 12 months because of irregular visit schedules (n = 150/794) were imputed and confidence intervals were adapted using Rubin’s formula [31, 32]. The other outcomes considered in this analysis were the time to the first malaria infection in infancy (defined by a positive RDT), the time to first diarrhoea episode and the time to first acute respiratory infection. Their association with the explanatory variables was assessed with a Poisson model [33, 34].

#### Explanatory variables

Two categories of explanatory variables were considered in the analysis:

- *Malaria exposure during pregnancy* The occurrence of any peripheral malaria infection, the number (0, 1 or >1) of peripheral malaria infections and the occurrence of placental malaria infection were considered in the analysis. To investigate if infant outcomes were associated with malaria infections occurring at a specific time during pregnancy, four time periods were defined starting from delivery; first 4 weeks before delivery, 4–12 weeks before delivery, 12–20 weeks before delivery and >20 weeks before delivery. As women were followed up from their first antenatal visit, malaria infection may have occurred before inclusion in the study and exposure to malaria during pregnancy was incompletely observed resulting in left-censoring. This missing information should not be coded as the absence of malaria infection, which may result in misclassification of true exposure but accounted for using statistical method for left-censoring [35]. In this analysis, multiple-imputation was used for measurement-error correction as detailed in “Appendix”.
- *Maternal characteristics and other potential confounders* Education level, mother age, gravidity, residential area, season, maternal HIV infection status and use of a bed net were considered in the analysis as potential confounders and included in all multivariate models. GA was also included as a covariate to account for the effect of preterm birth on infant growth.
- *Potential effect modifier* Maternal gravidity (primigravidae vs multigravidae) was also assessed as potential effect modifiers of malaria exposure.

This study was powered to assess the primary outcome and not for this secondary analysis. All analyses were performed using the open source statistical software R [36].

### Ethical approval

The study was approved by the institutional review boards of Mbarara University of Science and Technology, Uganda National Council for Science and Technology, and France’s “Comité de protection des personnes—Ile-de-France XI”. This study was registered with ClinicalTrials.gov, number NCT00495508.

### Role of the funding source

The sponsor of the study had no role in study design, data collection, data analysis, data interpretation, or writing of the report. The corresponding author had full access to all the data in the study and had final responsibility for the decision to submit for publication.

## Results

### Study population characteristics

**Fig. 1:**
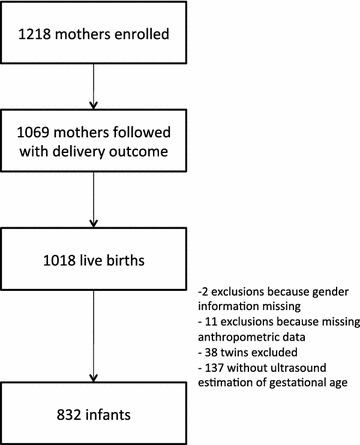
Flow chart

Of the 1218 women enrolled in the cohort, 60 % were enrolled before 20 weeks gestation, 1069 had delivery outcomes, and 1018 newborns were enrolled in the infant cohort. There were 832 (82 %) live-born singletons with a valid ultrasound assessment of GA included in this analysis (Fig. 1). The infants were followed for a median time of 12 months and 94 % had a follow-up ≥12 months. Mother and infant characteristics are summarized in Table 1 including the median (IQR) number of screens 21 (17–24) and doses of IPTp SP (68 % ≥2doses) provided to mothers. Infants who were included in this study were born to mothers with a greater education level (p = 0.001) and enrolled at a lower gestational age in the cohort (p < 0.001) when compared to those who were excluded. Peripheral malaria during pregnancy was observed in 198 (23 %) mothers and evidence of placental malaria was found in 15 (3 %) of the 490 placentas available. Mothers with placental malaria were included later in the study compared to those without placental infection (p = 0.002).

**Table 1:** Characteristics of the study population

Mother characteristics	
Median maternal age, year (IQR)	24 (21–27)
Primigravid, n (%)	311 (37)
Residence, n (%)	
Urban	446 (63)
Rural	386 (37)
Education level, n (%)	
No education	85 (10)
Primary	370 (45)
≥Secondary	377 (45)
HIV status, n (%)	
Positive	115 (14)
Unknown	94 (11)
Number of visits, median (IQR)	21 (17–24)
IPTp-SP, n (%)	
One dose	120 (15)
≥2 doses	563 (68)
Gestational age at inclusion (weeks), n (%)	
<15	145 (15)
[15 to <20−)	444 (45)
[20 to <24−)	224 (23)
≥24	173 (17)
Malaria exposure	
Placental malaria, n (%)	15 (3)
No malaria	626 (76)
1 episode	161 (19)
>1 episode	37 (4)
Malaria infections by gestation age (weeks), n (number at risk)	
<15	12 (120)
[15–20)	109 (630)
[20–24)	80 (822)
≥24	218 (986)
% of time ITN is used, median (IQR)	88 (56–96)
*Infant characteristics*	
Preterm delivery, n (%)	48 (6)
Gestational age at birth, media (IQR)	40 (39–41)
Small for gestational age, n (%)	140 (21)
Caesarean section, n (%)	
n (%)	124 (16)
% with HIV infection	21
Female, n (%)	452 (54)
Breastfeeding	
Number started, n (%)	808 (97)
Median duration, months (IQR)	4.6 (6.0–7.7)

### Infant growth

Of the 794 children with weight data, 141 (19 %) were SGA at birth. In adjusted analysis without including malaria variables, weight gain at 12 months was lower in girls, infants born by vaginal delivery, infants born to mothers infected with HIV and in those born SGA, while height gain was greater in boys, preterm infants and in those born to mothers with higher education level (Table 2).

**Table 2:** Factors other than malaria exposure during pregnancy associated with infant growth or morbidity

Variable	Weight growth	Height growth	Malaria^a^	Diarrhoea^a^	Acute respiratory infection^a^
Mother age^b^	0.01 (−0.01 to 0.03)	0.00 (−0.08 to 0.07)	0.97 (0.89–1.07)	0.98 (0.94–1.03)	0.98 (0.95–1.01)
Mother education level^c^					
Primary	0.01 (−0.25 to 0.28)	1.50 (0.51 to 2.49)	2.34 (0.53–10.39)	1.10 (0.63–1.93)	1.77 (1.16–2.77)
≥Secondary	0.21 (−0.07 to 0.49)	2.01 (0.97 to 3.04)	1.97 (0.41–9.54)	0.83 (0.45–1.52)	1.53 (0.98–2.39)
Residence area^d^	−0.05 (−0.20 to 0.11)	0.27 (−0.34 to 0.88)	1.31 (0.59–2.90)	0.79 (0.55–1.14)	1.21 (0.95–1.54)
Mother HIV status	−0.22 (−0.45 to 0.02)	−0.60 (−1.49 to 0.30)	2.06 (0.54–7.82)	1.62 (1.00–2.62)	0.86 (0.60–1.24)
Parity^e^	0.14 (−0.05 to 0.32)	0.18 (−0.54 to 0.90)	0.83 (0.34–2.14)	0.96 (0.63–1.69)	0.76 (0.57–1.00)
Mode of delivery^f^	0.33 (0.11 to 0.55)	−0.13 (−1.00 to 0.74)	1.63 (0.53–5.05)	1.04 (0.58–1.85)	1.02 (0.71–1.36)
Average time with net during pregnancy^g^	0.09 (−0.13 to 0.31)	0.25 (−0.66 to 1.16)	0.87 (0.30–2.47)	0.83 (0.52–1.34)	0.98 (0.71–1.36)
Duration of breastfeeding^h^	−0.18 (−0.52 to 0.16)	−0.68 (−1.96 to 0.60)	1.00 (0.95–1.05)	1.01 (0.98–1.03)	0.99 (0.97–1.00)
Child sex	−0.53 (−0.68 to −0.38)	−1.47 (−2.06 to −0.88)	3.69 (1.47–9.29)	1.10 (0.77–1.56)	0.82 (0.65–1.03)
Pre-term delivery	−0.05 (−0.35 to 0.26)	1.75 (0.52 to 2.97)	0.96 (0.22–4.17)	0.75 (0.33–1.72)	0.72 (0.43–1.21)
Small for gestational age	−0.16 (−0.35 to 0.03)	−0.07 (−0.81 to 0.66)	0.53 (0.15–1.78)	1.85 (1.25–2.74)	1.10 (0.82–1.47)
Season^i^	–	–	0.78 (0.37–1.69)	1.16 (0.80–1.68)	0.95 (0.75–1.20)

^a^Relative risk with 95 % confidence interval

^b^Per 10 years (centered)

^c^≥Secondary level or primary level versus no education

^d^Compared to mother living in town

^e^Primipare versus multipare

^f^C section versus vaginal delivery

^g^Weeks

^h^Weeks

^i^Rainy versus dry season

**Fig. 2:**
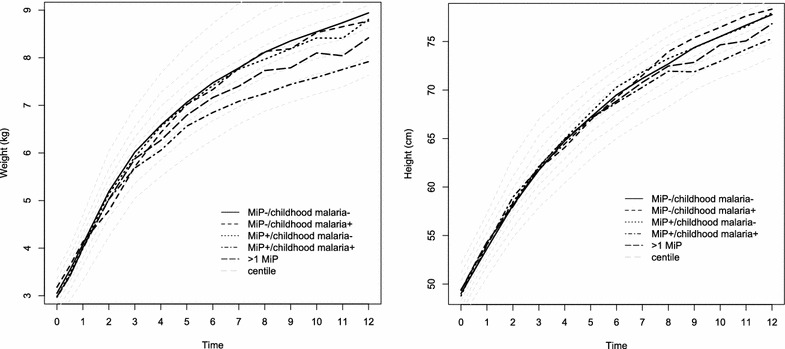
Weight and height growth with centiles for infants according to their exposure to malaria infection during pregnancy (MiP) (not exposed, exposed to MiP and exposed to >1 MiP) and to the occurrence of any malaria infection during their first year of life (childhood malaria)

A complex relation between malaria infection during pregnancy and growth was observed (Fig. 2). Overall, infants born to mothers exposed to >1 episode of MiP had significantly impaired height and weight gains when compared to those born to mothers not infected during pregnancy (respectively −2.71 cm, 95 % CI −4.17 to −1.25 and −0.42 kg, 95 % CI −0.76 to −0.08 in multivariate analyses). Infants born to mothers with placental malaria had significantly impaired weight gain (−0.65 kg, 95 % CI −1.16 to −0.13). There was no significant interaction between MiP and maternal gravidity (p = 0.7 and 0.1, respectively).

**Fig. 3:**
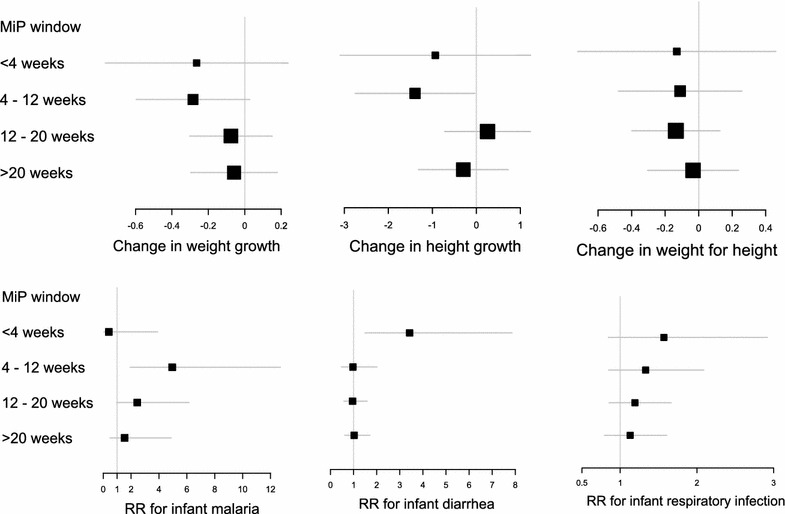
Association between the timing of malaria during pregnancy (counted backward from delivery) and clinical outcomes in infant

As displayed in Fig. 3 (upper panels), the risk of height and weight growth restriction was maximal for malaria infections occurring in the 12 weeks prior to delivery (respectively, −1.39, 95 % CI −2.76 to −0.03 and −0.28, 95 % CI −0.60 to 0.03, p = 0.07 for malaria infections occurring between 4 and 12 weeks before delivery) and declined the earlier in pregnancy the infection was detected and treated. A single infection promptly and effectively treated before 20 weeks without parasite reappearance had no significant modifying effect on infant weight growth (+0.03, 95 % CI −0.27 to 0.33, p = 0.9).

### Infant malaria

During their first year of life, 45 (5 %) children experienced at least one malaria episode, 416 (51 %) at least one acute respiratory infection and 177 (22 %) at least one diarrhoea episode. Of the 45 children with malaria only 7 had more than one infection but analysis of malaria was restricted to first occurrence. There was a significant positive association between the risks of diarrhoea and acute respiratory infection (p < 0.001) and a borderline association between the risks of malaria and of diarrhoea or acute respiratory infection (respectively p = 0.08 and p = 0.09). Only infections with *Plasmodium falciparum* were observed. The risk of malaria increased linearly over the first year of life (test for non linear trend: p = 0.8) and was higher in girls compared to boys (Table 2). The risk was similar between infants born to mother treated with quinine and those born to mother treated with AL (p = 0.2). A higher risk of malaria infection in infants was consistently found across the different variables used for malaria exposure; the risk was increased threefold in infants born to mother who had peripheral malaria (RR 2.97, 95 % CI 1.37–6.42) and more than tenfold in those born to a mother with placental malaria (10.42, 95 % CI 2.64–41.10). The risk of malaria was maximal when pregnancy malaria infection was confirmed (and treated) 4–12 weeks before delivery (Fig. 3, top right panel). Of note, in an analysis restricted to the subset of infants born to mothers who had MiP, those born to mothers with placental malaria were at increased risk of malaria during infancy (RR 8.04, 95 % CI 1.53–42.22) with wide confidence intervals due to the small numbers of placenta positive cases. There was no significant interaction on the log scale between peripheral malaria during pregnancy and maternal gravidity (p = 0.6). The small number of placental infections precluded meaningful interaction analysis.

### Infant morbidity

Regarding acute respiratory infection, there was a greater risk of infection in children of mothers who experienced peripheral malaria (RR 1.32, 95 % CI 1.02–1.71) or placental malaria (RR 1.92, 95 % CI 0.96–3.84, p = 0.06). No interaction was detected either with maternal gravidity (p = 0.2) or malaria timing (Fig. 3).

Lastly, the risk of diarrhoea was mainly increased in children born SGA (Table 2). An increased risk of infection was observed in children of mothers who experienced >1 malaria infection during pregnancy (RR 1.93, 95 % CI 1.02–3.66 for >1 MiP compared to no infection) or placental malaria (RR 2.25, 95 % CI 0.79–6.45, p = 0.12). The risk of diarrhoea was mainly increased when MiP occurred in the 4 weeks before delivery (3.48, 95 % CI 1.50–8.03).

## Discussion

In this study, microscopic malaria during pregnancy, more than one peripheral malaria infection or placental malaria infection, were associated with various impaired infant health outcomes during the first year of life, with greater adverse consequences when MiP was confirmed late in pregnancy.

A major association of malaria infection during pregnancy observed in this study was an increased risk of malaria during infancy, confirming previous reports [8–12, 14]. While most studies have used placental infection as a surrogate for malaria exposure during pregnancy, this study found that the association was consistently observed using other surrogates for malaria exposure during pregnancy. It should however be noted that infants born to a mother with placental malaria were at far more risk even compared to infants born to a mother who had peripheral malaria during their pregnancy but no placental infection. An increased risk of infant malaria was also observed when maternal malaria occurred within the last 12 weeks before delivery. This result conforms with previous evidence showing increased infant mortality and malaria morbidity associated with maternal malaria at the end of pregnancy [3, 13, 14]. The unexpected increased risk of malaria observed in girls may be due differences in the malaria prevention provided to girls and boys and calls for further investigations. A clear limitation of this analysis is the lack of measures of the actual individual exposure to malaria (i.e. local malaria transmission) although it has been shown that the association between placental malaria and infant malaria persist even after adjusting for environmental exposure to malaria [10]. As a consequence, it is not possible to rule out that the associations between malaria during pregnancy and infant outcomes observed in this study could be due to a common exposure of mothers and offspring to higher malaria transmission. This limitation does not detract from the underlying message of the importance of preventing maternal malaria and of explaining to these mothers that their child has a high risk of malaria so they can avoid delays in seeking treatment when the child is unwell.

Detailed studies have related the number and timing of malaria infection in pregnancy to the impact on low birth weight [24, 37, 38]. In this analysis, infants born to mothers who were exposed to ≥1 infection of MiP or placental malaria were at higher risk of impaired growth during the first year of life, which is consistent with results from two previous studies showing that MiP affects growth beyond the in utero period [6, 7]. In addition, in contrast to multiple or late malaria infections during pregnancy, a single infection occurring early in pregnancy and well treated with an efficacious regimen was not significantly associated with impaired growth during infancy. This highlights the importance of offering highly efficacious anti-malarials early in pregnancy to reduce the harmful effects of MiP. Mothers who had signs of placental infection were enrolled late in this cohort, possibly preventing the chance to detect a treatable peripheral malaria infection earlier in pregnancy or by chance really had malaria infection late in their pregnancy. This reinforces the benefit of systematic anti-malarial treatment of mothers who did not receive antenatal IPTp during their pregnancy. In this context the utility of RDT to detect peripheral parasitaemia, probably of low density, in the presence of placental malaria is questionable. Quantitative PCR techniques in these cases would allow a more comprehensive understanding of this dilemma reported frequently in the literature from African studies but such techniques are not readily available for the women who need them [39]. It should however be noted that this study does not allow a proper evaluation of the beneficial effect of frequent screening by RDT or of IPTp.

The consequence of MiP for the newborns in this study extended beyond the in utero period confirming results from Benin [15], the only other study that has examined this. In Mbarara, malaria infections were systematically screened with RDT at each visit and biologically confirmed with a BS. As a result it is unlikely that respiratory or diarrhoea symptoms were non-specific signs of undiagnosed malaria. In addition the association between MiP and respiratory infections or diarrhoea in the first year of life were independent of the mothers’ HIV status and other characteristics. The rates of respiratory infection and diarrhoea observed in this study were low compared to those observed in other settings likely because of the intensive follow-up during pregnancy reducing rates of placental malaria and because of the active care provided to the infants [40, 41]. Nevertheless the increased morbidity observed has important public health consequences such as an increased risk of death, hospitalization and drug prescriptions. The exact mechanism of the materno-fetal immune response leading to this increased infectious morbidity is important although there are few studies with accurate data on timing of MiP and longitudinally followed mothers and infants to contribute. An excellent summary of available studies providing evidence for the pathophysiological relationship between malaria in infancy in relation to MiP hypothesizes reduced antibody transfer in placental MiP and differences in the immune tolerance effects possibly due to HLA-G polymorphisms as underlying mechanisms but also acknowledges this information is incomplete [42]. Inference suggests that these changes in immunity due to MiP may be generalized and affect or interact with infant immune responses to non-malaria infection although evidence for this is currently sparse [15]. While an important strength of this study was the accurate determination of GA using gold standard ultrasound dating, several limitations should also be acknowledged. First, ultrasound dating was not available for all mothers. As a result some infant–mother pairs had to be excluded from this analysis, which might have resulted in some bias and lack of power. As the initial basis for the cohort was the assessment of malaria treatment for women in the 2nd and 3rd trimester, only a limited number of women were enrolled very early in pregnancy. Therefore, the effect of early malaria infection during pregnancy could not be assessed accurately from this data. Although accounting for left censoring with multiple imputation is expected to reduce bias, it should be emphasized that this method works under the assumption that missing data are non-informative which may not be true. Moreover, some confounders were not accurately measured and surrogate markers had to be used. For instance, only maternal HIV status or the time spent under a net were available. Nevertheless, the observed associations between MiP and infant morbidities suggests that these results are robust despite some possible residual confounding, and are important from a public health perspective.

## Conclusion

This study shows that, despite active screening and treatment, and IPTp for the majority of women, MiP, particularly late pregnancy infection, was associated with impaired infant growth and with both malaria and non-malaria infections during the first year of life. Prevention of MiP in pregnancy by efficacious treatment has the potential to reduce the significant burden of adverse consequences for mothers and infants.

## Appendix: Multiple imputation correction for incompletely observed malaria exposure [43]

At each time period T_i_, let X be the true MiP status indicator: X = 1 if a malaria infection was detected during T_i_ and X = 0 if no malaria infection was detected AND the observation was complete. R indicates if the observation was complete (R = 1) or not (R = 0) and W is the observed but possibly mismeasured MiP status. X was considered as missing whenever W = 0 and R = 0. Missing X were imputed using standard multivariate imputations by chained equations algorithm with W and the other covariates available. The number of imputations was increased to 50. For each imputed set, a regression model was fit and results were combined using Rubin’s formula [31].
